# Enhancing the Anti-Tumor Efficacy of NK Cells on Canine Mammary Tumors through Resveratrol Activation

**DOI:** 10.3390/ani14111636

**Published:** 2024-05-30

**Authors:** Tingting Zhu, Shengzi Jin, Danning Tong, Xingyao Liu, Yun Liu, Jiasan Zheng

**Affiliations:** 1 College of Veterinary Medicine, Northeast Agricultural University, Harbin 150030, China; ztt895731877@163.com (T.Z.); b210601011@neau.edu.cn (S.J.); 13394545588@163.com (D.T.); xingyaoliu@126.com (X.L.); 2 College of Animal Science and Veterinary Medicine, Heilongjiang Bayi Agricultural University, Daqing 163000, China

**Keywords:** canine mammary tumor, NK cells, resveratrol, immunotherapy, apoptosis

## Abstract

**Simple Summary:**

Mammary gland tumors are prevalent in canines, posing significant threats to their health and survival. With the limited efficacy of traditional treatments due to the high heterogeneity of these tumors, there is a growing focus on exploring new therapeutic avenues. Natural Killer cell-based immunotherapy has emerged as a promising approach in anti-tumor treatment, prompting research into optimization strategies. Resveratrol, a natural polyphenol compound, shows potential in regulating Natural Killer cell immune activity. However, the specific impact of Resveratrol-activated Natural Killer cells on canine mammary tumors and their therapeutic potential remain unclear. This study investigated the therapeutic potential of Resveratrol-activated Natural Killer cells in treating canine mammary tumors. Using various assays, including wound healing and flow cytometry, the study examined the impact of Resveratrol-pretreated Natural Killer cells on tumor cells in vitro and in a mouse model. The results demonstrate that Resveratrol enhances Natural Killer cells’ ability to induce tumor cell death and inhibit tumor growth, migration, and invasion. Moreover, Resveratrol boosts Natural Killer cell recruitment to other immune cells in the body. Overall, Resveratrol shows promise in enhancing Natural Killer cell-mediated anti-tumor effects, suggesting a potential avenue for optimizing immunotherapy for canine mammary tumors and improving treatment efficacy.

**Abstract:**

In order to explore the therapeutic effect of Resveratrol (Res)-activated Natural Killer (NK) cells on canine mammary tumors, this study employed a range of assays, including wound healing, colony formation, Transwell, flow cytometry, and Western blot experiments, to investigate the impact of Res-pretreated NK cells on canine mammary tumor cells in vitro. Additionally, a tumor-bearing mouse model was utilized to further analyze the therapeutic effects of Res-pretreated NK cells in vivo. The results showed that Res enhances the capacity of NK cells to induce apoptosis, pyroptosis, and ferroptosis in canine breast tumor cells, while also augmenting their influence on the migration, invasion, and epithelial-mesenchymal transition of these cells. Furthermore, pretreatment of NK cells with Res significantly amplified their inhibitory effect on breast tumor growth in vivo and promoted tumor tissue apoptosis. Additionally, Res enhanced the recruitment of NK cells to other immune cells in the body. In summary, Res has been shown to enhance the anti-breast-tumor effect of NK cells both in vitro and in vivo, offering a new avenue for optimizing immunotherapy for canine breast tumors.

## 1. Introduction

With the improvement of living standards, the number of companion animals kept by people has gradually increased, with canines being the most common type of companion animal. There has been a growing awareness of scientific breeding practices, leading to significantly extended lifespans for pets. However, tumors have emerged as a major threat to the life and health of elderly dogs. Traditional treatment approaches have primarily relied on surgical resection. Yet, malignant tumors often pose challenges, exhibiting high recurrence and metastasis rates, along with poor prognosis [1]. Moreover, due to the highly heterogeneous nature of canine mammary tumors, the efficacy of widely used chemotherapeutic drugs is limited, and their use can lead to substantial side effects [2]. Consequently, there is an urgent need for novel therapeutic approaches, and the advent of immunotherapy has brought renewed hope for cancer treatment [3,4].

The Natural Killer cell is a vital component of the body’s natural immune system and plays a critical role in the body’s defense against tumors. NK cell-based immunotherapy has emerged as a promising approach for treating malignant tumors, due to NK cells’ rapid and effective cytotoxicity against cancer cells without the need for prior sensitization. NK cells induce tumor cell death by releasing cytotoxic particles containing perforin and granzyme, as well as by releasing chemokines and cytokines that coordinate other immune cells [5,6,7,8]. Despite the notable clinical success of NK cell therapy in treating hematological tumors, its efficacy against solid tumors has been inconsistent [9,10,11]. To enhance NK cell therapy, strategies such as cytokine administration, immune checkpoint inhibitors, cell-engaging agents (BiKEs or TriKEs), and genetically modified NK cells (such as CAR-NK cells) have been developed to boost NK cell recognition and activation, thereby amplifying their cytotoxic effects [12]. In recent years, NK cell-based immunotherapy has gained increasing importance in anti-tumor therapy. The expansion and activation of NK cells in vitro can enhance their anti-tumor effects, underscoring the clinical significance of optimizing NK cell-based immunotherapy protocols for better outcomes in cancer treatment.

Res is a natural compound and antioxidant known for its diverse biological activities, including anti-tumor, anti-viral, anti-inflammatory, and anti-bacterial effects [13,14]. Recent studies have shown that Res can enhance the expression of activating receptors on NK cells [15]. The activation of NK cells by Res exhibits a notable bidirectional effect based on concentration levels: low doses activate NK cells and promote their proliferation, while high doses inhibit NK cell activation [16,17]. However, the specific impact of Res-activated NK cells on target cells and their therapeutic potential against solid tumors remain unclear. In this study, we assessed the anti-tumor effects of Res-pretreated NK cells on canine breast tumor cells both in vitro and in vivo in order to provide a new reference for the clinical treatment of canine breast tumors.

## 2. Materials and Methods

### 2.1. Cell Culture

**Figure 1:**
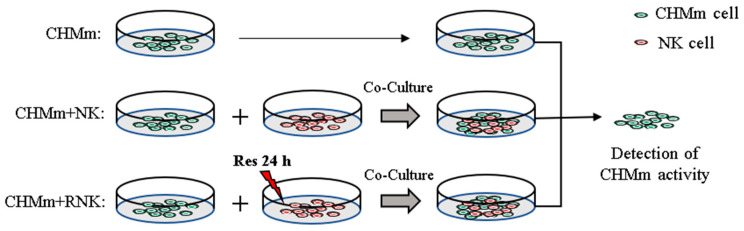
Cell experiment method flowchart.

CHMm cells (cells were isolated from the primary lesions of a 12-year-old female mongrel dog with breast tumor) were gifted by the Laboratory of Veterinary Surgery, University of Tokyo, Japan. NK cells were purchased from the Chinese Biomedical Laboratory Cell Repository. The NK cells were cultured in Alpha-modified Eagle’s medium (αMEM) containing 12.5% fetal bovine serum (FBS), 12.5% donor equine serum, 0.02 mM folic acid, 0.1 mM β-Mercaptoethanol, 0.2 mM Inositol, and 1% penicillin/streptomycin. CHMm cells were cultured in Dulbecco’s Modified Eagle Medium (DMEM) containing 10% fetal bovine serum (FBS) and 1% penicillin/streptomycin. All cells were cultured in a constant temperature cell culture incubator with 5% CO_2_ at 37 °C. The cell co-incubation method is shown in Figure 1.

### 2.2. CCK-8 Assay

NK cells were plated in a 96-well plate, and new media containing different concentrations (0, 2.5, 5, 7.5, 10, 12.5, 15, and 20 µM) of Res were added, followed by incubation for 24 h. Then, the medium was removed, and 10 µL of cell counting kit-8 (CCK-8) solution was added to each well for a 90-min incubation. The absorbance value at 450 nm per well was measured using a microplate reader to determine cell activity.

### 2.3. Cytotoxicity Assay

CHMm cells were plated in a 96-well plate and cultured in a 5% CO_2_ incubator at 37 °C for 12 h until all cells adhered to the bottom of the plate. The medium was removed, and new media containing Res-pretreated (0, 2.5, 5, 7.5, 10, 12.5, 15, and 20 µM) NK cells were added and incubated for 4 h. Then, the lactate dehydrogenase (LDH) content in cells in each group was detected according to the lactate dehydrogenase activity detection kit.

### 2.4. Annexin V-FITC Apoptosis Detection

CHMm cells were seeded in a 6-well plate, at a concentration of 1 × 10^5^ cells/well, and treated with NK cells and Res-pretreated NK cells for 4 h. After treatment, the cells and cell debris suspended in the culture medium were gently washed away with PBS. The CHMm cells were then collected into centrifuge tubes, using EDTA-free trypsin. The cells were stained according to the instructions of the Annexin V-FITC/PI kit. The fluorescence signal in the cells was collected using a BD FACSCelesta Flow Cytometer (Becton, Dickinson and Company, Franklin Lakes, NJ, USA), and the data were analyzed using FlowJo 10 software.

### 2.5. Clonogenic Assay

CHMm cells were harvested during the logarithmic growth phase and counted after trypsin digestion, and then 800 cells were seeded in each well of a 6-well plate. The cells were cultured at 37 °C for 6–8 days, with the medium changed every 2 days until most of the individual clones had grown. When the cell number exceeded 50, the cells were co-cultured with NK cells (at a concentration of 0 μM) and RNK cells (at a concentration of 7.5 μM) for 4 h each. After the co-culture period, the liquid was discarded, the cells were rinsed with PBS, and fresh medium was added. The cells were further cultured for 6–8 days, fixed with 4% paraformaldehyde solution at room temperature for 30 min, washed 3 times with PBS, and stained with 1 mL of crystal violet dye at room temperature for 20 min; the dye was then discarded, and the cells were washed 3 times with PBS and allowed to dry. Finally, pictures of each well were taken using a digital camera.

### 2.6. Wound-Healing Assay

CHMm cells were harvested during the logarithmic growth phase and counted after trypsinization, and then 8 × 10^5^ cells were seeded in 6-well plates. The cells were cultured in a 37 °C cell culture incubator for 24 h to allow for attachment. After 24 h, a line was drawn perpendicular to the well plate, using a 10 μL pipette tip. The cells were then washed with PBS and co-cultured with NK cells (Res, 0 μM) and RNK cells (Res, 7.5 μM) for 4 h. After the co-culture period, the liquid was discarded, and the cells were rinsed with PBS. Fresh medium containing 2% FBS was then added to the wells. The cells were further cultured in the cell culture incubator and stained, observed, and photographed at appropriate time points.

### 2.7. Transwell Cell Migration Assay

CHMm cells were harvested during the logarithmic growth phase and counted after trypsin digestion, and then 5 × 10^4^ cells/well were seeded in the upper chamber of the Transwell inserts. The cells were resuspended in 200 μL of complete culture medium and allowed to adhere to the wall. NK cells and RNK cells were then added to the wells at concentrations of 0 μM and 7.5 μM, respectively, and co-cultured for 4 h. Afterward, the medium was removed, and the upper chamber was gently washed with PBS. Following this, 200 μL of complete medium was added to the upper chamber, and 600 μL of 20% FBS complete medium was added to the lower chamber. The cells were then incubated for an additional 24 h. After incubation, the medium in the upper chamber was removed, and the chamber was washed three times with PBS. The cells were fixed with 4% paraformaldehyde solution at room temperature for 20 min; then, the solution was discarded, and the chamber was washed three times with PBS. The cells were stained with crystal violet for 15 min and then rinsed three times with PBS; and, after drying, images of each well were captured.

### 2.8. Cytofluorimetric Analysis

Cells were fixed in 4% paraformaldehyde for 30 min at room temperature and then permeabilized with 0.25% Triton X-100 for 15 min at room temperature. Subsequently, cells were blocked using PBST containing 1% BSA and 5% goat serum for 1 h. Next, cells were incubated overnight at 4 °C with primary antibodies against E-cadherin. Following this, cells were incubated with the appropriate secondary antibody for 1 h at room temperature. After mounting with DAPI-containing anti-fluorescent quencher, extensive washing was performed between each step. Images were then captured using fluorescence microscopy and analyzed.

### 2.9. Western Blotting Analysis

CHMm cells were lysed using lysis buffer containing protease inhibitors, and the proteins were subsequently extracted. Protein concentration was determined using the BCA protein concentration assay kit. The proteins were then denatured by adding loading buffer and boiling. A loading amount of 20 µg protein in each group was separated by 10% SDS-PAGE, and the separated protein was transferred to a Nitrocellulose membrane. After sealing at 37 °C for 1 h with 5% skim milk, primary antibodies targeting the following proteins were added and incubated overnight at 4 °C: β-actin (1:10,000), GAPDH (1:3000), Tubulin (1:1000), Bax (1:1000), Bcl-2 (1:1500), Bax-XL (1:1000), C-Caspase-3 (1:1000), GSDMD (1:1000), Caspase-1 (1:1000), NLRP3 (1:1000), Cytochrome C (1:1000), GPX4 (1:1000), vimentin (1:1000), β-catenin (1:1000), Wnt-3 (1:1000), E-Cadherin (1:1000), and N-Cadherin (1:1000). After the removal of the primary antibody, the corresponding secondary antibody was added and incubated at room temperature for 2 h. Finally, the target bands were developed using ECL luminescent liquid in the exposure instrument, and the protein bands were quantitatively analyzed using ImageJ 1.52 software.

### 2.10. Animal Studies

Twenty-one 6-week-old female BALB/c-nu nude mice were purchased from Liaoning Changsheng Experimental Technology Co., Ltd. (Changchun, China). The housing environment was maintained at a temperature of 27 °C ± 1 °C, a relative humidity of 50% ± 12%, and subjected to a 12 h light/dark exposure cycle. Adequate ventilation was ensured throughout the breeding process, and the environment was regularly disinfected. This study was performed strictly according to the recommendations of the Guide for the Care and Use of Laboratory Animals of the Ministry of Health, China. All animal welfare and experimental designs for this study were approved by the Northeast Agricultural University Animal Ethics Committee (#NEAU-2023–03-0146–1).

After one week of adaptive rearing, nude mice were subjected to subcutaneous tumor-bearing experiments. Well-growing CHMm cells were harvested and digested into a single-cell suspension using 0.25% trypsin. The CHMm cells were resuspended in an appropriate amount of phenol red-free Matrigel, and the cell concentration was adjusted to 1 × 10^7^ cells/mL. The CHMm cells were transferred to a sterile surgical site at low temperature, and 100 μL of the cell suspension was drawn into a 1 mL sterile syringe. The skin at the injection site was disinfected, and the cell suspension was injected into the subcutaneous fat pad of the right axilla. After the injection, the syringe was slowly withdrawn. Tumor volume at the injection site was then measured every 2 days. Cellular immunotherapy was initiated when the tumor volume reached 100 mm^3^.

CHMm tumor-bearing mice were randomly divided into three groups: the CHMm group (treated with PBS), the CHMm+NK group (treated with NK cells at a concentration of 1 × 10^7^), and the CHMm+RNK group (treated with RNK cells at a concentration of 1 × 10^7^). Immunotherapy was administered via peritumoral injection, with injections given every 3 days for a total of 3 treatments. Throughout the experiment, tumor volume and mouse weight were recorded every 2 days. After the completion of the treatment regimen, the mice were euthanized, and tumor tissue samples were collected for analysis.

### 2.11. Flow Cytometric Analysis

The prepared tumor cell suspensions and spleen cell suspensions of each group were divided into two groups for staining. One group was labeled with CD11b and F4/80 antibodies, while the other group was labeled with CD3, CD4, CD8α, and IFN-γ. The samples were incubated at 4 °C for 30 min in the dark, washed twice with PBS, and filtered through a 200-mesh screen. Finally, the fluorescence signals of the cells in each group were collected using the BD FACSCelesta Flow Cytometer instrument and analyzed using FlowJO 10 software.

### 2.12. Histological Staining and Imaging

The tumor tissues and visceral tissues were fixed in a 4% formaldehyde buffer solution for 24 h. The tissue blocks were embedded in paraffin using the paraffin embedding method and cut into 4 μm thick sections using a microtome. Tissue sections were then subjected to hematoxylin and eosin (H&E) staining using the H&E Stain Kit (Beijing Solarbio Science & Technology Co., Ltd., Beijing, China), following the manufacturer’s instructions. Finally, the images were observed using the Nikon Eclipse CI microscope and captured using the NIKON DS-U3.

### 2.13. IHC Analysis

The paraffin-embedded tumor tissue was sliced into 4 μm thick sections using a microtome, and the tissue antigens were then retrieved using a microwave method. A 3% hydrogen peroxide solution was employed to block endogenous peroxidase activity. BSA was added dropwise for blocking, and the blocking continued for 30 min. Next, the primary antibody specific to the detection index was applied to cover the tissue sections and incubated overnight at 4 °C in a humidified chamber, and no primary antibody was added to the negative control group. The dilution of each index is as follows: GPX4 (1:100), BCL-2 (1:100), BAX (1:1000), Caspase 3 (1:50), Ki67 (1:100), and VEGF (1:100). Rabbit secondary antibody was labeled (1:200) for 50 min at room temperature, followed by counterstaining of the nuclei with hematoxylin dye. Finally, the images were observed using the Nikon Eclipse CI microscope and captured using the NIKON DS-U3.

### 2.14. TUNEL Analysis

The paraffin-embedded tumor tissue was sectioned into 4 μm thick slices using a microtome. These sections were then dewaxed and hydrated, followed by dewaxing with xylene and a series of gradient ethanol washes (100%, 95%, 90%, 80%, and 70%) for 3 min each. Next, the sections were treated with Proteinase K working solution at 37 °C for 15–30 min. Subsequently, the sections were stained using the TUNEL Cell Apoptosis Detection Kit (Beijing Solarbio Science & Technology Co., Ltd., Beijing, China), and cell nuclei were counterstained with DAPI. Finally, an anti-fluorescein quencher was added dropwise, and the sections were observed using a fluorescence microscope.

### 2.15. Statistical Analysis

The data are presented as means ± standard deviation from a representative experiment conducted in triplicate. The date was plotted using GraphPad Prism 9. Statistical analysis involved an unpaired Student’s *t*-test for two groups and one-way analysis of variance (ANOVA) for more than two groups, utilizing SPSS version 22.0 software. A *p* < 0.05, *p* < 0.01, and *p* < 0.001 were considered statistically significant.

## 3. Results

### 3.1. Effect of RES on NK Cell Activity and Target Cell Toxicity

**Figure 2:**
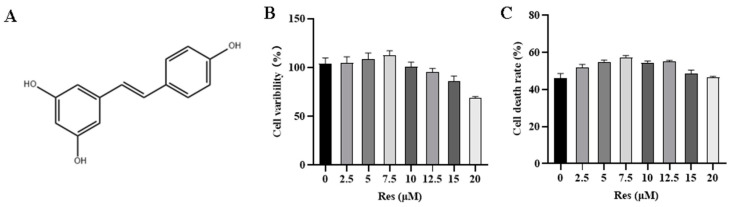
Effects of different concentrations of Res on NK cell activity. (**A**) Res molecular structure, (**B**) cck8 detects the effect of Res on NK cell activity, and (**C**) LDH release experiment to detect the effect of Res on the activity of NK cells killing target cells.

The proliferation ability of NK cells is the foundation of their anti-tumor effects. The effect of Res on NK cell activity was assessed using the CCK8 assay. The results, presented in Figure 2B, indicate that the survival rate of NK cells increases with Res treatment when the pretreatment concentration is below 7.5 μM. However, when the Res pretreatment concentration exceeds 7.5 μM, the proliferation of NK cells is inhibited. Subsequently, CHMm cells were used as target cells to evaluate the effect of Res pretreatment on the cytotoxicity of NK cells. As depicted in Figure 2C, the killing toxicity of NK cells increased with the Res concentration when the pretreatment concentration was below 7.5 μM. Notably, at 7.5 μM Res, there was a significant enhancement in NK cells’ killing toxicity.

### 3.2. Res Pretreatment Enhances the Ability of NK Cells to Induce Apoptosis in CHMm

**Figure 3:**
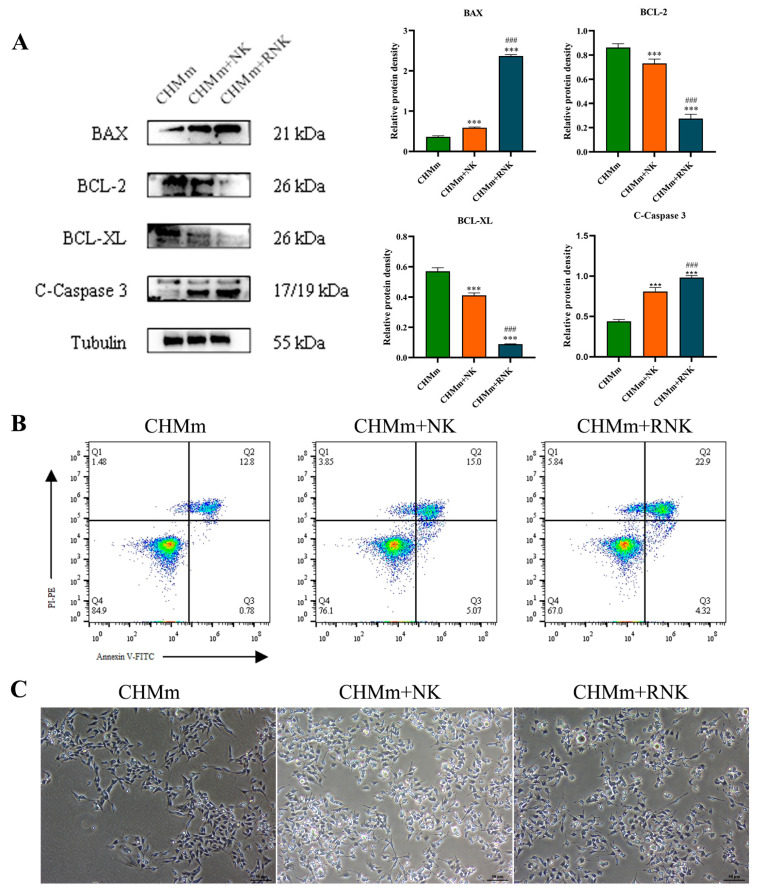
Effect of Res pretreatment of NK Cells on CHMm cell apoptosis. (**A**) Expression levels of apoptosis-related proteins in CHMm cells. (**B**) Flow cytometric analysis of CHMm cell apoptosis rate. (**C**) Observation of apoptotic bodies under a light microscope (bar = 50 μm). *** *p* < 0.001 compared with CHMm group; ### *p* < 0.001 compared with CHMm+NK group.

Inducing apoptosis in cancer cells by NK cells is a crucial aspect of NK immunotherapy. This study investigated the impact of Res on the NK cell-induced apoptosis of CHMm cells by examining apoptosis-related proteins and the cell apoptosis rate. The Western blotting analysis (Figure 3A) showed that, in the CHMm+NK group, the expression levels of pro-apoptotic proteins C-Caspase 3 and BAX significantly increased (*p* < 0.001) compared to the control group, while the levels of anti-apoptotic proteins BCL-2 and BCL-XL significantly decreased (*p* < 0.001). Compared to the NK group, the CHMm+RNK group exhibited an extremely significant increase (*p* < 0.001) in C-Caspase-3 and BAX protein levels, with reduced BCL-2 and BCL-XL levels (*p* < 0.001). The apoptosis detection results (Figure 3B) showed a significantly increased tumor cell apoptosis rate in the CHMm+NK group compared to the control group, and a further significant increase in the CHMm+RNK group compared to the CHMm+NK group. Furthermore, apoptotic bodies were observed under a light microscope, as shown in Figure 3C. In comparison with the control group, the number of apoptotic bodies in the CHMm+NK group significantly increased; moreover, compared with the CHMm+NK group, the CHMm+RNK group displayed a further increase in the number of apoptotic bodies. These findings suggest that Res pretreatment of NK cells can enhance their ability to induce apoptosis in breast tumor cells.

### 3.3. Res Pretreatment Enhances the Ability of NK Cells to Induce Pyroptosis and Ferroptosis in CHMm

**Figure 4:**
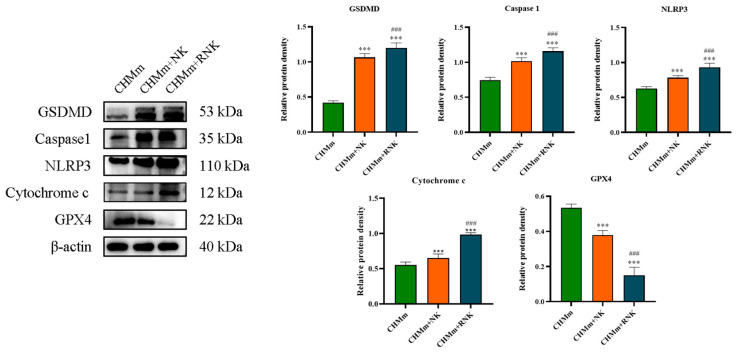
Effect of Res pretreatment on NK cells on CHMm cell pyroptosis and ferroptosis indicators. *** *p* < 0.001 compared with CHMm group; ### *p* < 0.001 compared with CHMm+NK group.

In addition to apoptosis, tumor cell pyroptosis and ferroptosis are key mechanisms for NK cells to fight tumors. This study detected the expression levels of key proteins of pyroptosis and ferroptosis and observed apoptotic bodies under a microscope to explore the effect of Res on NK cells during the induction of pyroptosis and ferroptosis in CHMm cells. The results of the Western blotting analysis are presented in Figure 4. In comparison with the control group, the protein expression levels of NLRP3, Caspase 1, Cytochrome C, and GSDMD in the CHMm+NK group significantly increased (*p* < 0.001), while GPX4 expression significantly decreased (*p* < 0.001). Furthermore, compared with the CHMm+NK group, the CHMm+RNK group exhibited an extremely significant increase (*p* < 0.001) in the expression of NLRP3, Caspase 1, Cytochrome C, and GSDMD proteins, along with an extremely significant decrease (*p* < 0.001) in GPX4 protein expression, indicating enhanced pyroptosis and ferroptosis induction in CHMm cells by Res-pretreated NK cells. 

### 3.4. Res Pretreatment Enhances the Inhibitory Effect of NK Cells on CHMm Cell Proliferation, Migration, and Invasion

**Figure 5:**
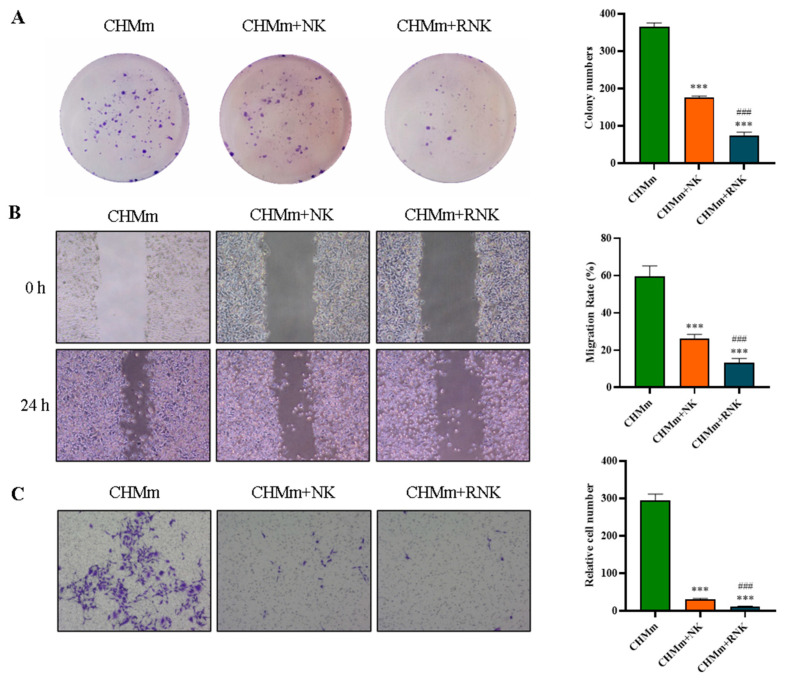
Effects of Res pretreatment of NK cells on the proliferation, migration, and invasion of CHMm cells. (**A**) Cell colony formation assay. (**B**) Wound-healing assay. (**C**) Transwell assay. *** *p* < 0.001 compared with CHMm group; ### *p* < 0.001 compared with CHMm+NK group.

In this study, we examined how Res-pretreated NK cells influenced the proliferation, migration, and invasion of CHMm cells using cell colony formation, wound healing, and transwell assays, the results are shown in Figure 5. Our findings revealed that Res significantly increased the inhibitory effect of NK cells on the proliferation, migration, and invasion abilities of CHMm cells. Specifically, the cell colony formation assay showed a remarkable decrease in cell colonies in the CHMm+NK group compared to the control group, indicating suppressed proliferation. Similarly, the wound-healing assay demonstrated a substantial reduction in cell migration rates in the CHMm+NK group, suggesting impaired migration ability. Furthermore, the transwell assay revealed a significant decrease in cell invasion rates in the CHMm+NK group, indicating reduced invasion ability. These results suggest that Res pretreatment enhances the ability of NK cells to inhibit the proliferation, migration, and invasion of CHMm cells, highlighting its potential as a therapeutic strategy against canine breast tumor.

### 3.5. Res Pretreatment Enhances the Inhibitory Effect of NK Cells on EMT of CHMm Cells

**Figure 6:**
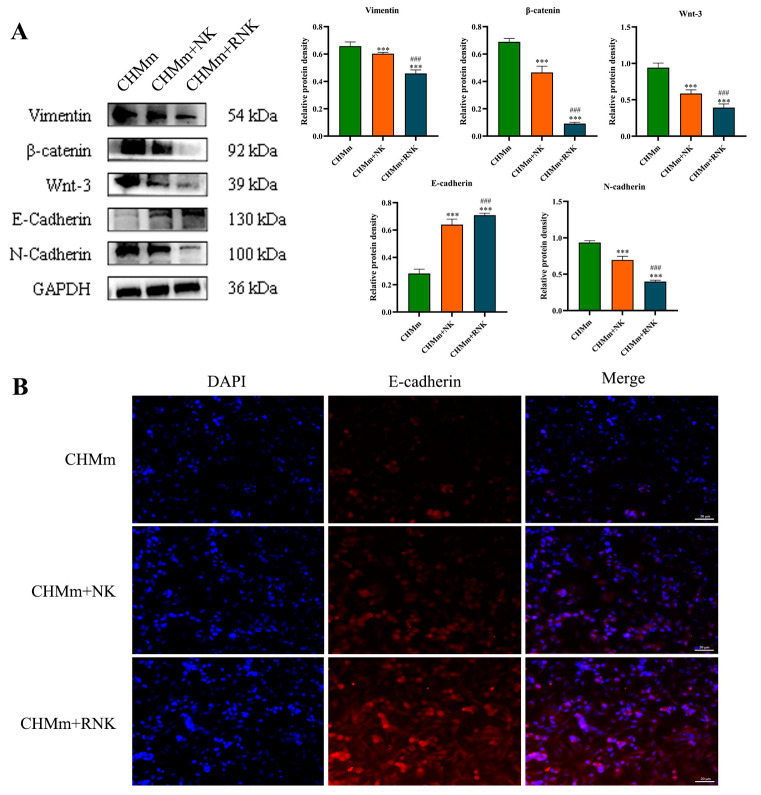
Effect of Res pretreatment NK on tumor cell EMT-related proteins. (**A**) Detection and analysis of CHMm cell EMT-related proteins. (**B**) Immunofluorescence detection of E-cadherin (bar = 20 μm). *** *p* < 0.001 compared with CHMm group; ### *p* < 0.001 compared with CHMm+NK group.

In order to investigate the impact of Res pretreatment on NK cells on the EMT of CHMm cells, this study employed Western blotting and immunofluorescence to assess the expression of proteins associated with breast tumor EMT. The results of the Western blotting are depicted in Figure 6A. In comparison to the control group, the protein levels of Vimentin, β-catenin, Wnt-3, and N-cadherin in the NK group were significantly reduced (*p* < 0.001), while E-cadherin expression was significantly increased (*p* < 0.001). Furthermore, when compared to the CHMm+NK group, the CHMm+RNK group exhibited an extremely significant reduction (*p* < 0.001) in Vimentin, β-catenin, Wnt-3, and N-cadherin expression, and an extremely significant increase (*p* < 0.001) in E-cadherin expression. E-cadherin was detected by immunofluorescence, and the results are shown in Figure 6B. The red fluorescence intensity of N-cadherin in the CHMm+NK group was significantly weakened compared to that of the control group (*p* < 0.001), and, similarly, the red fluorescence of the RNK group was also significantly decreased (*p* < 0.001) compared to that of the NK group. These findings suggest that Res-pretreated NK cells can effectively suppress the migration capability of breast tumor cells.

### 3.6. Res Enhances the Inhibitory Effect of NK Cells on Tumors in Tumor-Bearing Mice

**Figure 7:**
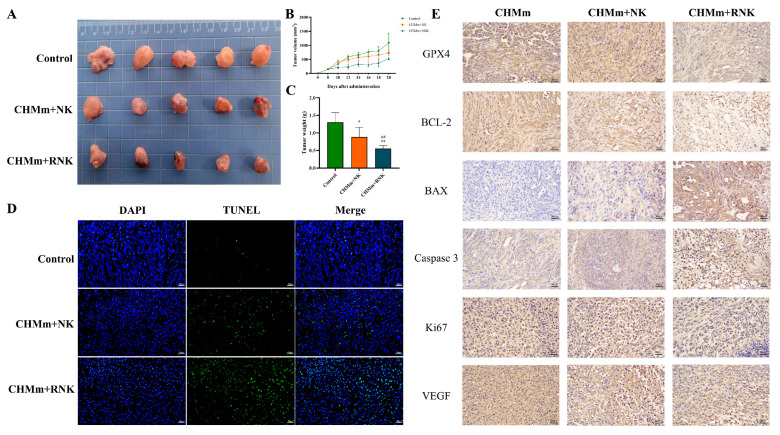
Tumor volume and weight of tumor-bearing mice. (**A**) Photograph of representative breast tumors in different groups. (**B**) Mean tumor volume of mice implanted with CHMm cells. (**C**) Weight of breast tumors in different groups. (**D**) TUNEL staining results of tumor tissue (bar = 50 μm). (**E**) Immunohistochemical staining results of tumor tissue (bar = 50 μm). This has been supplemented in the manuscript. * *p* < 0.05 compared with CHMm group; ** *p* < 0.01 compared with CHMm group; ## *p* < 0.01 compared with CHMm+NK group.

During the experiment, the tumor volume of control-group mice gradually increased over time. Treatment with NK cells resulted in an inhibition of tumor growth, and this inhibitory effect was more pronounced after treatment with Res-pretreated NK cells, as shown in Figure 7B. Visual observations and tumor weight measurements of the tumors in each group of mice after treatment are presented in Figure 7A,C. These results demonstrate that Res can significantly enhance the inhibitory effect of NK cells on tumor growth. 

The results of tumor HE identification in the control group were shown in Figure S1.

### 3.7. Res Pretreatment Enhances NK Cell Therapy to Induce Tumor Tissue Apoptosis and Ferroptosis

The impact of Res pretreatment on NK cell therapy-induced tumor cell apoptosis was assessed using TUNEL staining. The results are shown in Figure 7D, where TUNEL-positive cells are marked in green, and the nucleus in blue. Apoptotic cells in both the CHMm+NK group and the CHMm+RNK group significantly increased compared to the control group. Moreover, the apoptosis rate of the CHMm+RNK group was significantly higher than that of the CHMm+NK group. Additionally, IHC was used to analyze the expression of apoptosis and ferroptosis-related indicators in tumor tissues. The results are presented in Figure 7E. In comparison to the control group, both the CHMm+NK and CHMm+RNK groups exhibited a significant increase in the positive rates of Caspase 3 and BAX cells, while the positive rates of BCL-2 and GPX4 cells showed a significant decrease. Furthermore, when compared to the CHMm+NK group, the CHMm+RNK group displayed a significant increase in the positive rates of Caspase 3 and BAX cells in tumor tissue, along with a significant decrease in the positive rates of BCL-2 and GPX4. The results of immunohistochemical analysis were shown in Figure S2. These findings suggest that Res pretreatment significantly enhances NK cell-induced tumor tissue apoptosis and ferroptosis. 

### 3.8. Effect of Res Pretreatment of NK Cells on Proliferation and Metastasis Indicators in Breast Tumor Tissue

IHC was employed to assess Ki67 and VEGF, crucial indicators of malignant tumor proliferation and metastasis. The results are illustrated in Figure 7E. In comparison to the control group, both the CHMm+NK and CHMm+RNK groups exhibited a substantial reduction in the positive rates of Ki67 and VEGF cells in breast tumor tissues. Furthermore, when compared to the CHMm+NK group, the CHMm+RNK group displayed a further significant reduction in the positive rates of Ki67 and VEGF cells in breast tumor tissues. These findings indicate that Res pretreatment of NK cells can effectively inhibit the proliferation and migration of tumor tissue in vivo.

### 3.9. Res Enhances NK Cells and Enhances the Recruitment of Immune Cells in Mouse Spleen and Tumor Tissues

**Figure 8:**
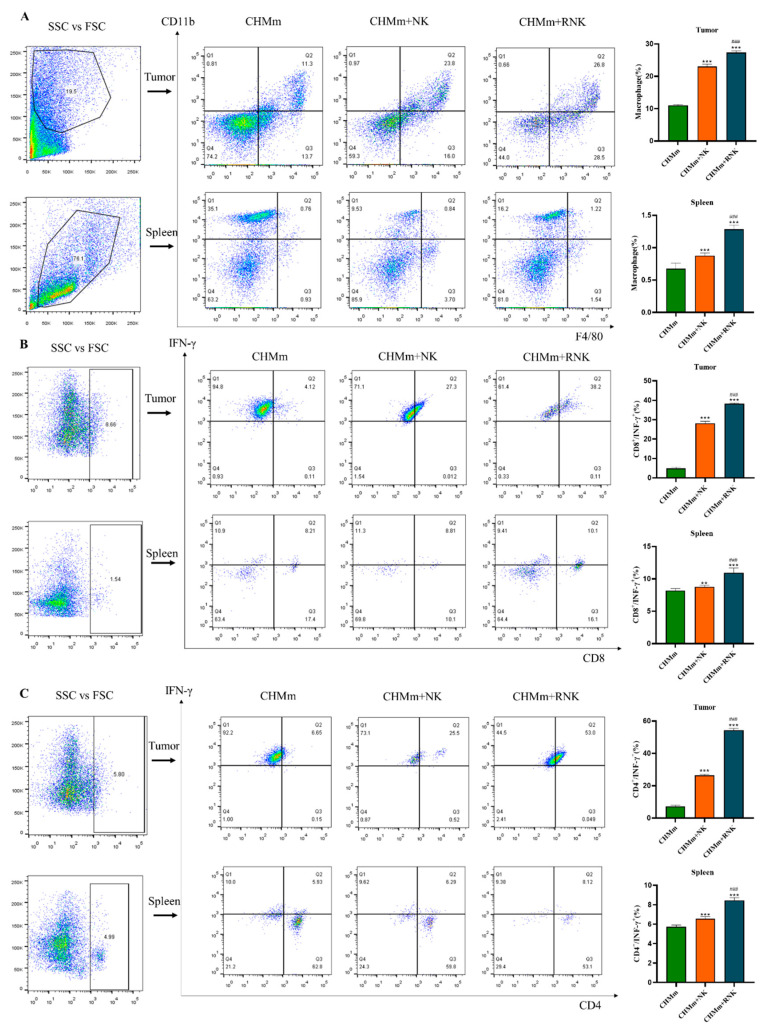
Flow cytometric analysis of immune cells in spleen and tumor tissue. (**A**) Infiltration of macrophage in tumor tissue and spleen. (**B**) Infiltration of CD8^+^ IFN-γ^+^ T cells in tumor tissue and spleen. (**C**): Infiltration of CD4^+^ IFN-γ^+^ T cells in tumor tissue and spleen. ** *p* < 0.01 compared with CHMm group; *** *p* < 0.001 compared with CHMm group; ### *p* < 0.001 compared with CHMm+NK group.

The immune effector mechanism primarily relies on immune cells in the body, with macrophages and T cells playing crucial roles as effector cells. Therefore, the presence of macrophages, CD4^+^ T cells, and CD8^+^ T cells in spleen and tumor tissues was analyzed using flow cytometry. The results of cell recruitment are depicted in Figure 8. Following treatment with NK cells and RNK cells, there was a significant increase in the positive rates of macrophages, CD4^+^ T cells, and CD8^+^ T cells in both tumor tissues and spleens. Furthermore, Res-pretreated NK cells exhibited a significantly enhanced recruitment of macrophages, CD4^+^ T cells, and CD8^+^ T cells in both tumor tissues and spleen compared to the NK cell group.

## 4. Discussion

Resveratrol, a polyphenolic natural compound, has been studied for its ability to regulate the energy metabolism and function of immune cells. Recent research has demonstrated that Res can enhance the expression of activating receptors on NK cells. Its effect on promoting NK cell activity shows a clear bidirectional relationship with concentration [15]. In this study, NK92 cells and CHMm cells were used as the subjects of investigation. It was observed that the Res treatment concentration of 7.5 μM had the most significant effect on promoting the proliferation of NK cells. Additionally, the cytotoxicity test revealed that NK cells treated with 7.5 μM Res exhibited the strongest killing effect on CHMm cells. These results indicate that an appropriate concentration of Res can significantly enhance the anti-tumor activity of NK cells. Consequently, 7.5 μM Res was chosen for subsequent experiments involving NK cell treatment.

The primary objective of immunotherapy is to induce tumor cell death for therapeutic purposes. Currently, there are three main mechanisms through which immunotherapy can induce cell death: apoptosis, pyroptosis, and ferroptosis. Apoptosis is a well-studied process in immunotherapy, particularly in NK cell immunotherapy, which has demonstrated anti-tumor effects in various types of tumors [18,19,20]. When NK cells recognize target cells, they initiate cell apoptosis through pathways involving tumor necrosis factor (TNF), Fas ligand (FasL), and TNF-related apoptosis-inducing ligand (TRAIL). In this study, it was observed that NK cells can upregulate the expression of pro-apoptotic proteins such as C-Caspase 3 and BAX in breast tumor cells, while inhibiting the expression of anti-apoptotic proteins like BCL-2 and BCL-XL. These findings are consistent with previous research, supporting the notion that NK cell-induced tumor cell apoptosis contributes to its anti-tumor effects. Furthermore, pretreatment of NK cells with Res significantly enhanced the expression of tumor cell apoptosis-related proteins induced by NK cells, potentially augmenting its anti-tumor efficacy. The study also analyzed the effect of Res-pretreated NK cells on the apoptosis level of breast tumor cells using Annexin V-FITC/PI staining. The results revealed that Res-pretreated NK cells increased the apoptosis rate of tumor cells, further validating that Res pretreatment can enhance NK cell-mediated tumor cell apoptosis, thereby boosting its anti-tumor efficacy. Pyroptosis, a form of programmed necrotic cell death, has emerged as an important mechanism in tumorigenesis and the tumor immune microenvironment. Recent studies have indicated that NK cells can induce pyroptosis in tumor cells, thereby exerting anti-tumor effects [21,22]. The classical pyroptosis pathway involves the detection of pathogen-associated molecular patterns (PAMPs) and damage-associated molecular patterns (DAMPs) by pattern-recognition receptors, leading to the recruitment and activation of ASC to form the NLRP3 inflammasome. This complex then activates Caspase 1, which cleaves the linker region of gasdermin D (GSDMD) and exposes its N-terminal pore domain with pore-forming activity, resulting in cell swelling and eventual cell death. The current study investigated key proteins involved in this classical pyroptosis pathway, including Caspase 1, NLRP3, GSDMD, and Cytochrome C. The results indicated that NK cells can significantly increase the expression of pyroptosis-related proteins in tumor cells, with Res-treated NK cells further enhancing this effect. These findings suggest that Res pretreatment can enhance the NK cell-mediated induction of pyroptosis in breast tumors. Additionally, ferroptosis is recognized as an effective mechanism in anti-tumor activity. Studies have shown that interferon-gamma (IFN-γ) secreted by immune cells can induce ferroptosis in tumor cells, and Res has been demonstrated to activate and increase the expression of IFN-γ [23,24]. Therefore, this study examined the expression of the key ferroptosis-related protein, GPX4. The results revealed that pretreatment with Res enhanced the expression of GPX4 protein in breast tumor cells by NK cells, indicating that Res-pretreated NK cells can increase the sensitivity of breast tumor cells to ferroptosis, thus enhancing the anti-tumor activity of NK cells.

Canine mammary tumors represent a highly heterogeneous disease, with approximately 50% classified as malignant tumors [25,26]. The epithelial–mesenchymal transition (EMT) process in tumor cells is a crucial indicator of the transition from static primary tumors to aggressive and metastatic malignancies. During EMT, epithelial cells lose polarity and cell–cell adhesion, gain migratory properties, and increase invasiveness, transitioning into mesenchymal-like cells. Thiery JP et al. observed significant activation of the EMT process in patients with metastatic breast tumor, resulting in cancer cells acquiring a fibroblast-like and spindle morphology. This process is characterized by a loss of the epithelial marker E-cadherin, along with increased expression of the mesenchymal markers Vimentin and N-cadherin [27,28,29]. Recent studies have highlighted the impact of the EMT process on the anti-tumor efficacy of NK cells, particularly emphasizing the role of E-cadherin expression in NK cell-mediated cytotoxicity [30] Additionally, the Wnt/β-catenin signaling pathway is known to be significantly activated in highly invasive metastatic breast tumor cell lines, with an overactive pathway correlating with poor clinical prognosis [31,32,33,34]. In light of this, the current study investigated the expression of EMT and Wnt/β-catenin pathway-related proteins in CHMm cells following co-incubation with NK cells. The findings revealed that NK cells induced an increase in the expression of Vimentin and N-cadherin in CHMm cells, while the expression of E-cadherin, Wnt-3, and β-catenin decreased, suggesting an inhibition of the EMT process. Notably, NK cells pretreated with Res further enhanced this inhibitory effect. These results indicate that Resveratrol pretreatment can enhance the inhibitory effect of NK cells on the EMT process of CHMm cells, thereby suppressing the metastatic and invasive potential of CHMm cells. Furthermore, the study assessed the effect of Res-pretreated NK cells on the migration and invasion ability of CHMm cells using cell colony formation assay, wound-healing assay, and transwell assay. The results consistently demonstrated that Res pretreatment enhanced the migratory inhibition and reduced the invasion ability of CHMm cells by NK cells. These findings collectively suggest that Res pretreatment can augment the anti-metastatic properties of NK cells against CHMm cells.

Cell experiments are highly valuable for preliminary screening and our understanding of the molecular and cellular mechanisms of cancer. However, they do have certain limitations when it comes to tumor treatment research. Therefore, in this study, a mouse model bearing CHMm cells was established to evaluate the effect of Res pretreatment on NK cells and their anti-tumor efficacy in vivo. The results demonstrated that NK cells exerted a significant inhibitory effect on the growth of canine mammary tumors. Moreover, the anti-tumor effect of NK cells was further enhanced after Res pretreatment, resulting in a stronger suppression of tumor growth. Furthermore, NK cells induced tumor cell apoptosis and ferroptosis, which remained evident in solid tumors, consistent with the findings from in vitro studies. Tunnel staining of tumor tissue further confirmed that Res pretreatment of NK cells significantly improved their anti-tumor efficacy. Proliferation of solid tumors is a critical factor for evaluating the effectiveness of tumor treatment, with Ki-67 and VEGF serving as key indicators that reflect tumor proliferation and are crucial for assessing the prognosis of breast tumors [35,36,37,38]. In this study, Res pretreatment markedly enhanced the inhibitory effect of NK cells on Ki-67 and VEGF protein expression in tumor tissues, suggesting that Res-pretreated NK cells can effectively inhibit tumor cell proliferation and angiogenesis. Consequently, the in vivo experimental results are consistent with those from the in vitro experiments, underscoring that pretreating NK cells with Res can significantly augment their anti-tumor efficacy.

NK cells can collaborate in anti-tumor effects by recruiting and activating T cells within the tumor microenvironment (TME). Activated NK cells provide a substantial amount of IFN-γ to prime T cells, and IFN-γ is crucial for inducing Th1 polarization. The expression of OX40 and B7 ligands on the surface of activated NK cells induces the proliferation of autologous T cells [39]. The cytokine IL-2 released by T cells also plays a role in NK cell activation [40]. Macrophages can also be recruited by NK cells to play a synergistic anti-tumor role in the tumor microenvironment, and cytokines play an important role in the crosstalk between NK cells and macrophages [41]. In the tumor microenvironment, macrophages release activating cytokines such as IL-12, IL-15, and IL-18, which alone or collaboratively promote NK cell survival, proliferation, and maturation and the production of the pro-inflammatory cytokines IFN-γ and GMCSF, further enhancing the anti-tumor cytotoxicity of NK cells. The IFN-γ produced by NK cells can induce the polarization of macrophages and play a synergistic anti-tumor effect. In addition, IL-10 secreted by macrophages can enhance the lytic activity of NK cells [42].

Numerous studies have highlighted that the interplay among NK cells, T cells, and macrophages within the TME plays a pivotal role in the anti-tumor immune response [43,44]. The findings from this study reveal that Res pretreatment of NK cells enhances the recruitment of mature macrophages in both the spleen and tumor microenvironment. Moreover, it increases the presence of CD4^+^ IFN-γ^+^ T cells and CD8^+^ IFN-γ^+^ T cells within the spleen and tumor microenvironment. These results suggest that Res-pretreated NK cells may augment the recruitment of macrophage, CD4^+^ IFN-γ^+^ T cells, and CD8^+^ IFN-γ^+^ T cells. The cooperative action of these immune cells helps to overcome immune suppression within the tumor microenvironment, thereby enhancing the anti-tumor effect of NK cells.

## 5. Conclusions

In summary, our study demonstrates that Res enhances the ability of NK cells to induce apoptosis, pyroptosis, and ferroptosis in canine breast tumor cells, while also augmenting their impact on the migration, invasion, and epithelial–mesenchymal transition of these cells. Moreover, in vivo experiments reveal that Res amplifies the inhibitory effect of NK cells on tumor growth, promotes tumor tissue apoptosis, and enhances the recruitment of NK cells to other immune cells. These findings validate the ability of Res to enhance the anti-breast-tumor effect of NK cells. Our study provides a crucial theoretical foundation for optimizing NK cell therapy and presents a novel treatment approach for the clinical management of canine breast tumors.

## Data Availability

All data used during the study appear in the submitted article.

## Supplementary Materials

The following supporting information can be downloaded at: https://www.mdpi.com/article/10.3390/ani14111636/s1, Figure S1: Breast tumor tissue sections of CHMm tumor bearing mice (bar = 50/100 μm); Figure S2: Results of immunohistochemical analysis.
