# Supplementary material for: Enhancing the Anti-Tumor Efficacy of NK Cells on Canine Mammary Tumors through Resveratrol Activation
Source: Animals (Basel). 2024 May 30;14(11):1636. doi: 10.3390/ani14111636 (PMC11171074; doi:10.3390/ani14111636)
Supplement: Supplementary file 1 [file animals-14-01636-s001.zip › animals-3033184-supplementary.pdf]

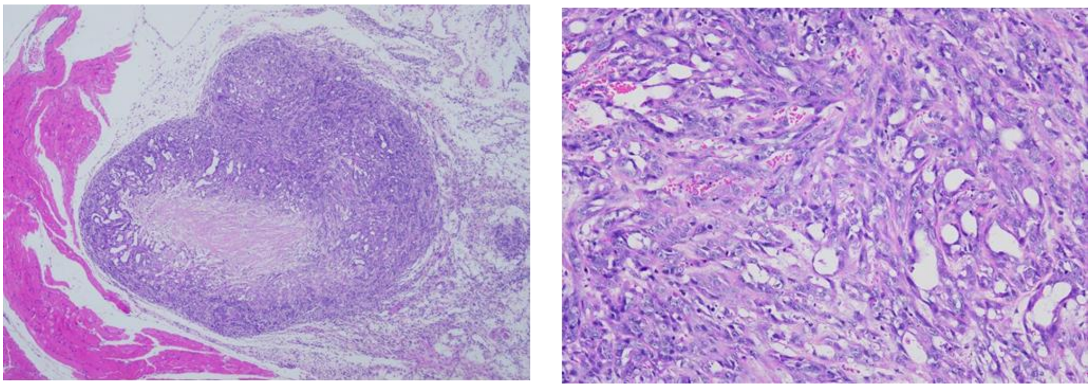

Figure S1. Breast tumor tissue sections of CHMm tumor bearing mice (bar=50/100 μm).

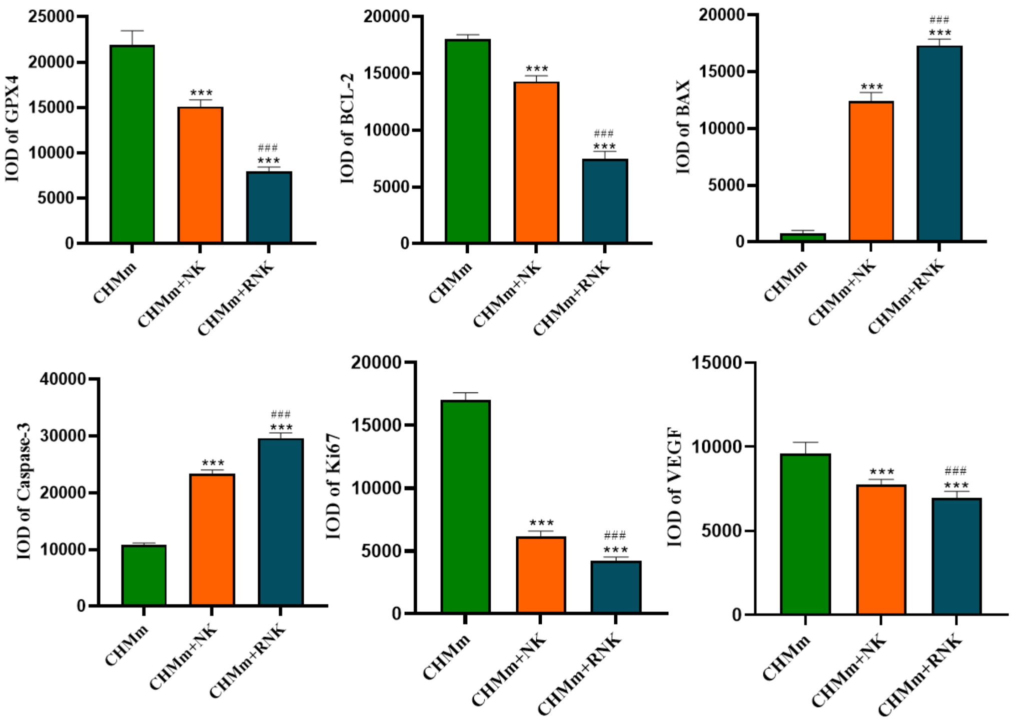

Figure S2. Results of immunohistochemical analysis.
